# Sensory Information and Encounter Rates of Interacting Species

**DOI:** 10.1371/journal.pcbi.1003178

**Published:** 2013-08-15

**Authors:** Andrew M. Hein, Scott A. McKinley

**Affiliations:** 1 Department of Biology, University of Florida, Gainesville, Florida, United States of America; 2 Department of Mathematics, University of Florida, Gainesville, Florida, United States of America; University of Texas at Austin, United States of America

## Abstract

Most motile organisms use sensory cues when searching for resources, mates, or prey. The searcher measures sensory data and adjusts its search behavior based on those data. Yet, classical models of species encounter rates assume that searchers move independently of their targets. This assumption leads to the familiar mass action-like encounter rate kinetics typically used in modeling species interactions. Here we show that this common approach can mischaracterize encounter rate kinetics if searchers use sensory information to search actively for targets. We use the example of predator-prey interactions to illustrate that predators capable of long-distance directional sensing can encounter prey at a rate proportional to prey density to the 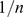 power (where 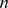 is the dimension of the environment) when prey density is low. Similar anomalous encounter rate functions emerge even when predators pursue prey using only noisy, directionless signals. Thus, in both the high-information extreme of long-distance directional sensing, and the low-information extreme of noisy non-directional sensing, encounter rate kinetics differ qualitatively from those derived by classic theory of species interactions. Using a standard model of predator-prey population dynamics, we show that the new encounter rate kinetics derived here can change the outcome of species interactions. Our results demonstrate how the use of sensory information can alter the rates and outcomes of physical interactions in biological systems.

## Author Summary

Encounters between individual organisms are an essential part of biology; in many sexually reproducing species, males and females must encounter one another in order to mate, pollinators must find flowers, and predators must locate prey before capturing and consuming them. Many species accomplish these tasks by actively searching for their targets using sensory information. Despite this, classical mathematical models used to predict the rate of encounters between searchers and their targets assume that searchers make movement decisions without using sensory information. Here we develop a mathematical framework for incorporating sensory information into searcher movement behavior to study how sensory response changes the relationship between encounter rate and target density. By comparing searchers that use sensory information to those that do not, we show that sensory response not only increases encounter rate, but that it also changes the form of the relationship between encounter rate and target density. By using sensory information, predators encounter prey at a rate that is less sensitive to changes in prey density when prey density is low. Our results demonstrate a strong connection between the usage of sensory information and the encounter rates that are so critical to survival and reproduction in nature.

## Introduction

Classical models of species interactions assume that encounters between individuals are governed by a process akin to mass-action; individuals move along random linear trajectories and encounter one another when they come within a critical distance [1], [2]. Under these assumptions, a searcher such as a predator or pollinator will encounter its targets at a rate that scales linearly with target density [1],[3]. The form of the function relating encounter rate to target density is essential; it affects both the dynamics and outcome of species interactions. The form of this relationship at low target density, in particular, strongly influences ecological and evolutionary dynamics in systems of interacting species by determining the degree to which a searcher can deplete limiting resources (e.g., [4]).

Recent work has extended the study of encounter rates to consider searchers that encounter targets probabilistically, destroy targets after encounters, search intermittently, and follow trajectories that are not linear [5]–[7]. Under a variety of circumstances, these models too predict linear scaling (for a list of conditions, see [6]). Yet, a vital assumption both of older and newer models is that the searcher moves independently of the locations of its targets. In the context of predator-prey interactions, this implies for instance that predators do not alter their movement behavior in response to sensory cues emitted by their prey.

Of course, the assumption that searchers move independently of targets is often made for mathematical convenience. The question is whether models that rely on this assumption capture the salient features of encounter rate kinetics in nature. Empirical studies have shown that inhibiting particular sensory modalities such as chemosensing or flow sensing can dramatically decrease search performance (e.g., [8]), and that sensory cues appear to influence both small-scale [9] and large-scale [10],[11] search behavior. While such studies more rigorously confirm the intuition that using sensory data should improve search performance, little is known about how sensing can influence the functional form of the relationship between encounter rate and target density. Here, we show that sensory response can have a dominant effect on the rate of encounters between searchers and their targets, not only by increasing mean encounter rate, but also by qualitatively changing the form of the relationship between encounter rate and target density.

Below we adopt the language and intuition associated with a predator searching for prey. We assume that the predator samples its environment for sensory cues passively emitted by prey, and adjusts its movement behavior according to explicit mathematical models presented here. This approach builds on a recently developed framework for modeling search decision-making [12] to model the flow of sensory information from prey to predators. We consider three scenarios: (1) *perfect sensing and response:* the predator can ascertain the precise locations of prey from the sensory data it receives and responds optimally, (2) *imperfect sensing and response:* the predator detects noisy scent signals emitted by prey and modulates its movement behavior in response, and (3) *purely random search:* the predator does not use sensory information to guide its movement decisions. We choose (1) and (2) in such a way that they represent upper and lower bounds, respectively, on the acquisition and use of information about prey positions. Our central finding is that there is a systematic shift away from a linear encounter rate function at both of these bounds, suggesting that the collection and use of sensory data may fundamentally alter encounter rate kinetics. We discuss the role of information in governing predator-prey encounter rates, but note that our general methodology could be applied to rates of encounters in other types of ecological interactions (e.g., between mates, competitors, mutualists). We propose that the linear encounter rate models that are typically used to model interaction rates may not correctly capture encounter rate kinetics at low target densities. If this is indeed the case, the most commonly used models of coupled population dynamics, food webs, competition, immune function, and many other systems, may mischaracterize the outcome and dynamics of species interactions.

## Methods

### Encounter rate and search behavior: Some definitions

Studies of biological search typically describe how the type of movement behavior used by a searching organism affects the time needed to encounter its first target 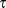, or the rate of target encounters 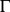. For consistency with past work, we define 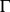 as the prey encounter rate of a single predator (e.g., prey per [predator hour], [6]). We assume that predator density is low enough that 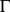 does not depend on the density of predators, and instead, depends only on the density of prey 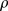. We define two encounter rate functions: the mean first encounter rate 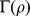, and the mean encounter rate after 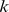 encounters 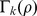. The latter is often referred to as the encounter rate associated with *destructive search* [6],[7], emphasizing that the activity of the searcher alters the target landscape. In past studies, the *non-destructive search* rate is often defined in terms of random variable 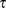 which represents the time required to find the first target. The empirical first encounter rate is then defined to be 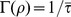 where 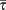 indicates an average over many searches.

### Framework for modeling movement decisions

To illustrate how sensory information can affect encounter rates, we consider an idealized model of a searching predator in a two-dimensional environment (we discuss search in three dimensions in Text S1). We assume that the predator moves at a constant speed 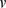 and that prey do not move, at least for the duration of the predator's search. This approximation applies to a wide variety of realistic predator-prey interactions (e.g., large terrestrial carnivores searching for grazing prey). Moreover, incorporating prey movement behavior significantly complicates analysis, for example by introducing the need for a game theoretic formulation of the searcher-target interaction [13]. In the following sections, we further assume that prey density is low, and that handling time is therefore negligible relative to search time. As in past approaches, the predator divides its search into two phases: a scanning phase and a movement phase [12],[14]. This intermittency reflects the observed tradeoff between locomotion and perceptual acuity (e.g., [15]), and the intermittent nature of sampling through major sensory modalities [16]. During the scanning phase, the predator collects sensory data 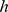, and encounters any prey within a radius 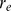 with probability one. During the movement phase, the predator moves a distance 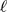 at an angle 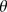. After moving, the predator re-enters the scanning phase and this process is repeated. Thus, the predator's movements consist of a set of movements with scanning phases in between. The process the predator uses to determine 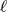 and 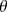 constitutes its search strategy.

When prey density is low, a predator will typically detect little or no signal and proceed with the information that the target is not likely to be nearby. When prey density is high, an increasing fraction of the landscape is covered by regions that are within the encounter radius 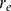 of a prey item. In this way, our modeling framework naturally captures a predator's behavioral transition between these two regimes. For example, if prey are distributed according to a Poisson process, a simple Poisson thinning argument shows that the probability that a given location is not within the encounter radius of a prey item is given by 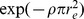. Thus, as prey density 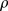 increases, there is a seamless transition from limited to perfect information about where the nearest prey is located.

#### Sensory signals and search behavior

To model predator search behavior, we generalize a recently developed framework for modeling search decision-making [12]. The framework has two essential features. First, the predator's movement behavior in the absence of any sensory data is modeled by an intrinsic movement distribution 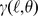. Second, the predator uses a decoding function to extract information from the sensory data it collects and modify its intrinsic movement behavior.

During the movement phase of the search, predator movements are modeled by drawing from the distribution
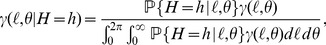
(1)where 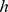 is the sensory data collected in the previous scanning phase, and 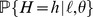 is the likelihood of observing sensory signal 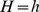, given that the target is a distance of 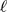 and angle 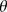 from the predator's current position. Rather than associating a deterministic action with a particular value of the signal 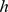, we model movement decisions as actions drawn from a probability distribution to capture the inherent variability in decision-making [17]. The intrinsic movement distribution can be interpreted as an evolved behavior that the predator uses in the absence of useful sensory information [18]. The decoding function, on the other hand, represents an evolved mechanism for interpreting and moving based on sensory input [12]. While 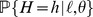 is formally a likelihood function, we refer to it as a decoding function to emphasize that it represents a means of interpreting and using signal data. As we show below, the three strategies we wish to consider can be framed by specifying appropriate decoding functions.

#### Perfect sensing and response

**Figure 1:**
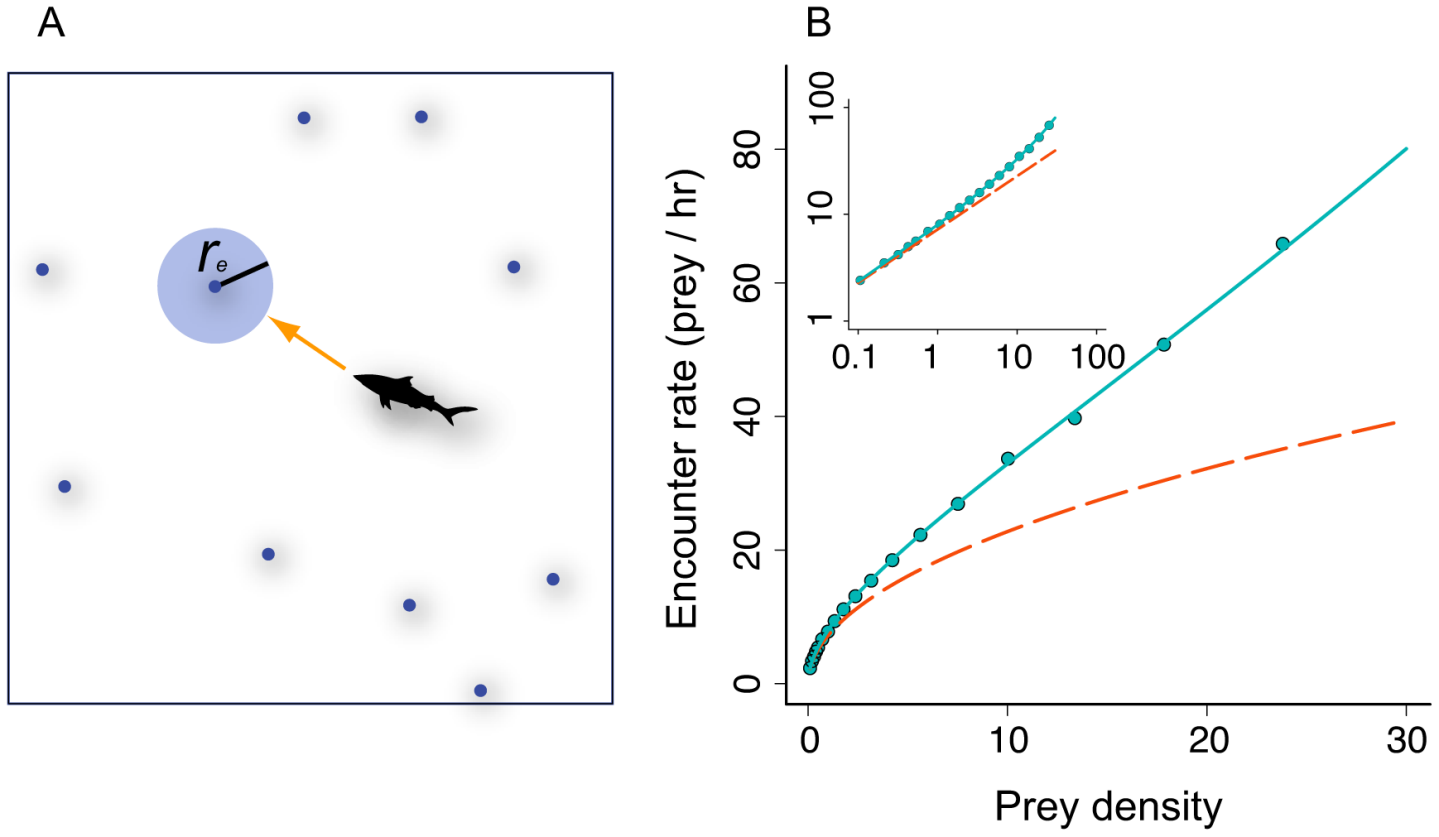
Encounter rate of a predator with perfect sensing and response. A) Predator with perfect sensing and response searching in a two-dimensional environment. After collecting sensory data, the predator moves along a linear trajectory toward the nearest prey and encounters the prey when it comes within a distance of 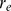. B) Mean encounter rate from simulations (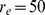 body lengths, 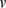 = 1 bl s^−1^, points show mean of 1000 replicates at each density). Prey distribution is randomly generated from a Poisson point process in each simulation. Solid cyan curve shows theoretical mean encounter rate (see text), which approaches 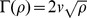 for low prey density (dashed red curve). Inset shows simulation data and theoretical curves with logarithmic axes for densities up to 100. For 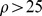, the typical distance between nearest prey is less than 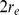 and predators begin to encounter prey frequently without having to search. In this and subsequent figures, density is expressed as prey per 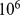 squared predator body lengths.

Suppose the predator detects sensory observation 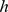 and, regardless of the value of 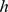, is able to perceive the precise locations of prey. Then the decoding function in equation (1) is a point mass at the location of the nearest prey. (Note that a “traveling salesman” solution to this problem could outperform such a greedy searcher, but is computationally intractable when the number of prey is not small.) In this case, movements are taken from the distribution 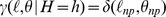, where 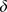 denotes the Dirac delta function and 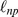 and 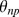 are the distance and angle between the predator's current position and the location of the nearest prey. In each movement phase, the predator moves along a linear trajectory from its current position to the position of the nearest prey (Fig. 1). The form of the intrinsic movement distribution is unimportant, so long as it satisfies certain technical mathematical requirements such as having continuous density with non-zero value at 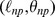.

When the predator moves directly from one prey to the next, it will encounter prey at a mean rate that is inversely proportional to the mean distance between prey, which we denote 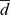. Assuming prey are distributed according to a Poisson spatial process, 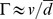, or equivalently 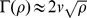. Formally, this calculation requires that prey are replenished and redistributed after each encounter and that there is no net decrease in prey density. It also assumes that the encounter radius is zero. To compute encounter rate when encounter radius is greater than zero – for example, when predators have some finite visual distance or striking distance – we observe that a predator must move an average distance of 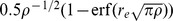 in order to reach the encounter radius of its nearest prey (see Text S1). It follows that the encounter rate is 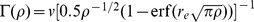 (Fig. 1B, solid cyan curve and points). When the density is such that the mean distance between targets is similar to 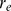, encounter rate changes linearly with prey density (Text S1). However, as density approaches zero, this function approaches 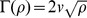 (Fig. 1B, dashed red curve). So unlike encounter rate models that assume predators move independently of prey, a predator with perfect sensing and response will encounter prey at a rate that is proportional to the square root of prey density when density is low. More generally, when predators search in an *n*-dimensional environment with low prey density, encounter rate 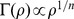 (Text S1).

#### Purely random search

Purely random search models have a long history, beginning with the models of Lotka [1]. Recently, there has been a resurgence of interest in random search behavior as the possible outcome of selection for maximal encounter rate with targets in the absence of useful sensory information. For example, studies of “Lévy walk” behavior have argued that searching organisms can increase the rate at which they encounter targets by moving according to random walk behavior in which the lengths of steps are drawn from a distribution with a power law tail [7],[19]. Theoretical results claiming that such Lévy walk behavior is an optimal search strategy and empirical results claiming that organisms perform Lévy walk-like behavior have been contentious (e.g., [5],[20],[21]). Nevertheless, many studies use both Lévy and non-Lévy random walks to model search behavior. We include purely random search behavior here because it is one canonical model used to derive encounter rates, regardless of whether searchers are assumed to have Lévy or non-Lévy behavior.

We note that it is possible to formulate a search behavior that does not rely on sensory data using the Bayesian framework of Equation (1) by assuming that the decoding function 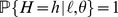 for all 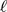 and 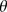. Each time a predator moves, it draws a step length and turn angle from the distribution defined by equation (1), which is just the intrinsic movement distribution 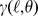 when the decoding function is uniform. In this interpretation of the purely random search scheme, predator movements may be independent of sensory signals in the environment for any of three reasons: (1) the predator cannot detect and/or neurally encode the signal, (2) the predator can detect the signal but cannot extract information from the encoded signal, or (3) the predator has the sensory and neural machinery for encoding and decoding signals, but does not use the information it gleans to make movement decisions.

#### Imperfect sensing and response

In addition to the predator with access to perfect information, and the predator with access to no information, we consider a predator with access to minimal sensory data and a minimal capacity for decision-making. We do this to demonstrate that even minimal information use can significantly alter the encounter rate function [12]. A case of search with minimal sensory information occurs in organisms that use noisy odor cues to locate resources in turbulent environments. Species like sharks, lobsters, and crabs routinely face such a challenge [8],[22]. We assume that the predator receives noisy scent signals that lack directional information and modulates its behavior accordingly. We refer to this as *signal-modulated* search behavior. In a given time interval *t_0_* the predator will encounter a number of detectable scent patches drawn from a Poisson distribution. The predator's mean rate of scent encounters depends on the distance to targets in its vicinity. We assume that all targets have the same intensity of signal emission and the rate of arrivals at a distance 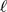 is given by a function 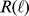. As in past approaches, we assume 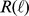 is given by the steady state solution to the diffusion equation describing the diffusion and dissipation of scent without advection (see Text S1, [12],[23]). In this case, the decoding function is given by the likelihood
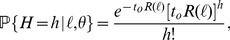
(2)where 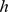 represents the number of detectable scent arrivals in some fixed amount of time *t_0_*.

This model of olfactory search behavior has two salient features. The first is that, because there is no directional information inherent in the signal, the predator always draws turn angles from the same distribution (Uniform on 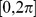), regardless of the signal it receives. Second, the predator has no memory of past movements or signal encounters. Such information could expedite search [23] but we do not explore the impact of memory because we wish to model a predator that uses signal data in a minimal fashion.

### Encounter rates in simulated searches

In addition to the analytical results described above, we used search simulations to compare the behavior of a predator that moves according to a purely random strategy to a predator with imperfect sensing and response. In both cases, we assume that the intrinsic movement behavior is described by a symmetric two-dimensional Pareto distribution. Because of the symmetry we can separately draw the turn angle 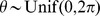 and the move length 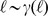, where 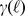 is the density of a Pareto random variable,
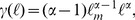
(3)

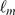
 is a minimum move length, and 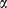 is a parameter that determines whether the walk is superdiffusive (superdiffusive for 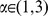). We use a Pareto distribution with a power law tail to model intrinsic movement behavior because it has been argued that such a distribution may have evolved as a statistical movement strategy for locating resources when sensory data are not useful [19]. In Text S1, we show that our qualitative results hold when predators move according to a diffusive random walk. As shown in past work [12], the use of sensory data to make movement decisions dominates intrinsic movement behavior so that the distinction between diffusive and superdiffusive intrinsic strategies becomes relatively unimportant.

In each simulation, we placed a single predator in a prey periodic environment (i.e., environment was a torus) and populated the environment with a Poisson number of prey with mean 600. The size of the environment was then scaled to achieve the desired prey density. In each scanning phase, 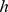 was sampled from a Poisson distribution with mean given by Equation (S2) (see Text S1) summed over all prey. In each simulation, the searcher was positioned at a random location and allowed to move through the environment until it came within a distance of 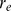 of a prey item during its scanning phase. We designated this an *encounter* and the prey was destroyed. For each strategy, we performed 1,000 simulations and recorded the time until the desired number of prey encounters was achieved. Predators were assumed to travel at a constant speed of one body length per second, which is a realistic speed for foraging predators.

We performed two sets of simulations. In the first, prey positions were generated using a Poisson point process. We then recorded the time required for the predator to encounter the first prey and used this to compute encounter rate 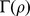. This is consistent with a scenario in which predators search for and capture a single prey item, and then cease to forage for a period of time, during which prey redistribute themselves in the environment. When predators encounter and destroy multiple prey in succession, they can create local zones of prey depletion. To determine whether the scaling of encounter rate is sensitive to such a local depletion effect, we allowed predators to encounter and destroy 32 prey items. We then computed 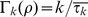, where 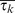 was the mean time required to encounter 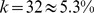 of the prey present on the environment. We chose 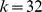 as a compromise between maximizing the number of targets encountered in a single search, while ensuring that this local depletion did not substantially change global target density. For reasons of computational efficiency, we wished to limit the mean number of targets in the environment to 600, and therefore chose 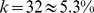 of targets as a reasonable compromise. Finally, to determine whether the scaling of the encounter rate depends on the distribution of targets, we generated prey distributions according to a highly clustered point process that we will call a *preferential attachment* model and repeated simulations to compute 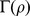 and 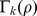 for 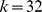. Briefly, 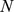 prey were generated by drawing from a Poisson distribution with mean 600. The size of the environment was then scaled to achieve the desired prey density. A fraction of the 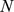 prey were chosen to act as *seed* points and placed uniformly at random on the space. The remaining prey were each assigned as *daughters* to one of the seed points iteratively with probability 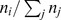, where 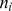 is the number of daughters around the *i*th seed point. Positions of daughters were assigned uniformly within a circle of radius 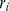 around the seed point, where 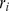 was chosen so that all clusters had the same local prey density.

Our primary goal was to characterize the form of the encounter rate function in the low prey density regime. We simulated predators exploring environments with prey densities ranging from 0.5–100 prey per 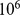 squared predator body lengths. All simulations were performed using Matlab. The lower limit of the prey density range was chosen based on realistic low prey densities encountered by predators in nature. For example, Serengeti lions experience densities of ungulate prey that can be as low as 0.3–10 prey per 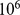 squared predator body lengths; snow leopards experience densities of their primary prey, blue sheep, as low as 7 prey per 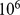 squared predator body lengths; and northern hawk owls capture and consume small rodents with densities as low as 40 prey per 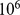 squared predator body lengths [24]–[26].

### Estimation of scaling regimes and exponents

As in previous investigations (e.g., [5]), we expected that 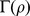 would be a linear function of 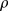 for the purely random predator. On the other hand, as shown above, the predator with perfect sensing and response has an encounter rate function with several scaling regimes in the range of densities that interest us: one in which encounter rate is proportional to 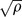, and one in which encounter rate is proportional to 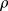. To accommodate these functional forms, we assumed that locally, the encounter rate can be described by a power function of the form 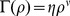. This allows for both linear and sublinear scaling. To determine whether simulated predators had multiple scaling regimes, we fitted (i) a single power function, (ii) a segmented function with two distinct scaling regimes, and (iii) a segmented function with three distinct scaling regimes. Prior to fitting, we log-transformed density and encounter rate data from search simulations. We used a recently developed statistical method for simultaneously estimating both the break points between distinct scaling regimes and the scaling exponents in each regime [27]. Briefly, this technique allows one to fit a piecewise regression model in which the locations of the break points in the piecewise function are fitted parameters in the model. This technique is particularly useful when fitting functions to data when there is no *a priori* knowledge of the precise transitions between regimes. We compared the fits models with a single regime to models with multiple regimes using 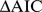. We computed 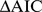 as the AIC value of the model with one scaling regime minus the AIC value of the best fitting model with two or three scaling regimes. Statistical analyses were conducted using the Segmented package [28] in R [29].

## Results

**Figure 2:**
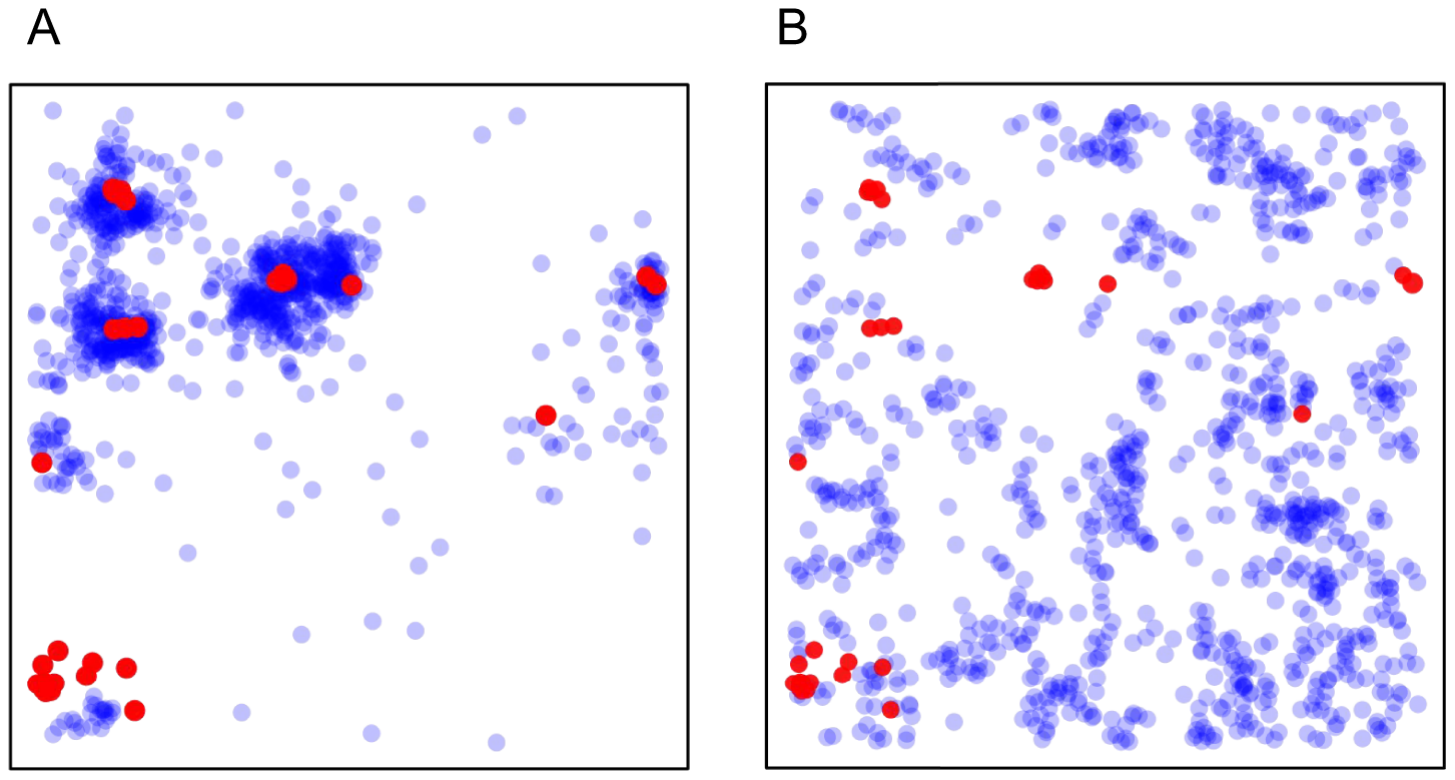
Scanning locations of signal-modulated and purely-random predators. Prey (red points) and locations where predator scans for prey (blue points) for A) signal-modulated and B) purely-random predators. Scan points are semitransparent so darker color indicates locations where predator has scanned more frequently. Data represent searches in which a predator made 1000 consecutive movements without destroying prey.

There is a dramatic difference between movement patterns of predators that use sensory data and those that do not. As is evident from Fig. 2, predators with imperfect sensing and response concentrate scanning effort near prey (Fig. 2A), whereas purely random predators scan roughly uniformly over the environment (Fig. 2B). In the study of animal search, concentration of effort near targets is known as area-restricted search (ARS). ARS is a canonical feature of search behavior in nature [30]. It is interesting that this seemingly complex behavior can emerge from an extremely reduced sensing and decision making process like the one modeled here [12].

**Figure 3:**
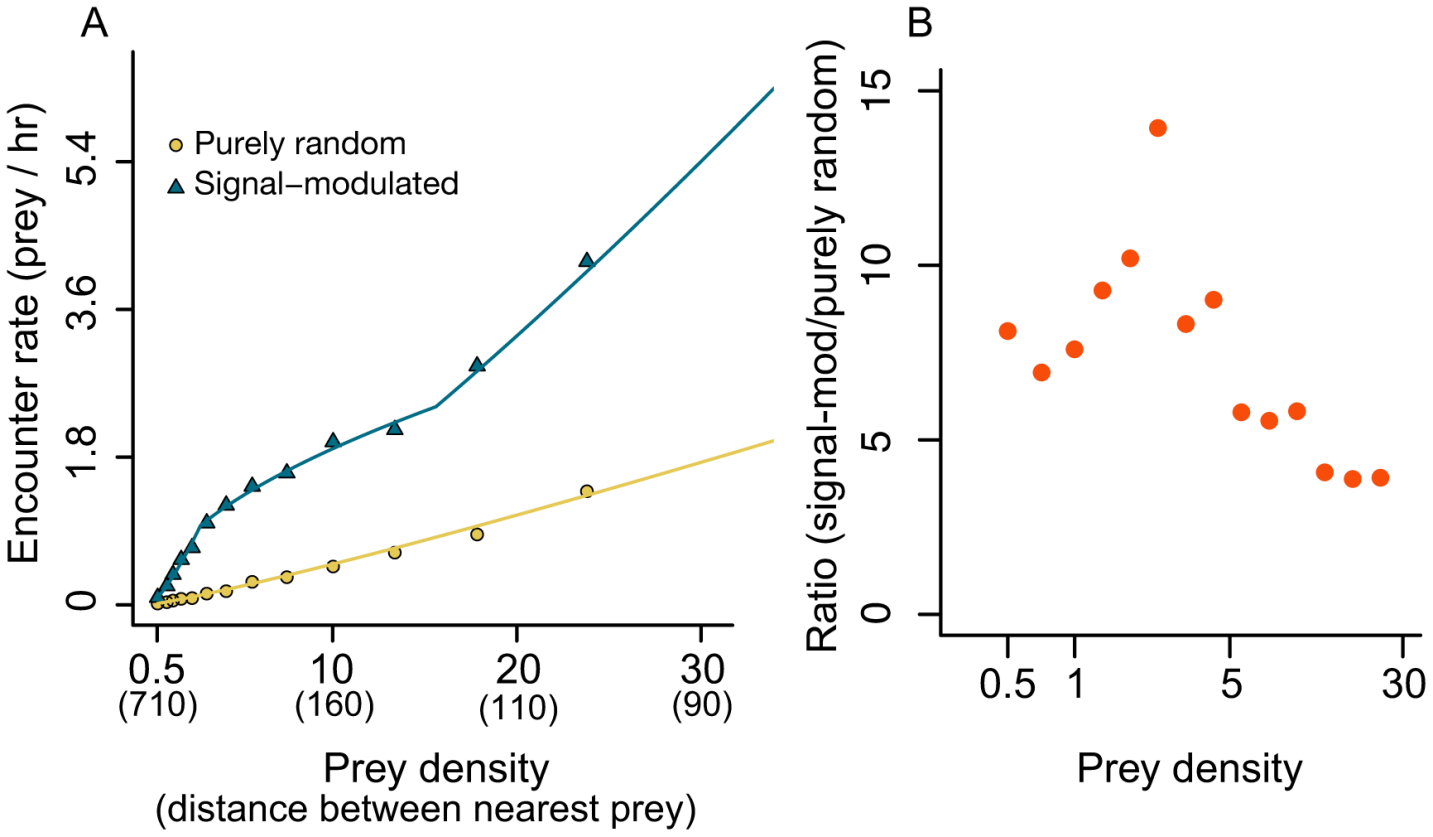
Encounter rates of purely random and signal-modulated predators. A) Purely random (yellow circles) and signal-modulated predators (blue triangles, *k* = 1) searching in uniform (Poisson) prey environment. Each point represents mean encounter rate from 1000 replicate simulations. In simulations shown, the following parameters were used: 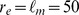 body lengths, 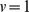 body length per second, *r_o_* = 500 body lengths, 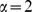. Scent emission rate at prey location was set to 100 (see Text S1). B) Ratio of encounter rates shown in A (rate of signal modulated predator divided by rate of purely random predator).

Signal-modulated predators perform ARS because they move short distances between scans when they receive strong sensory signals and move long distances when they measure weak signals [12]. This behavior improves search efficiency, but perhaps more importantly, it leads to a qualitatively different relationship between the encounter rate of signal-modulated predators and their prey (Fig. 3A). As expected from past work on random search [5],[6], purely random predators encounter prey at a rate that scales nearly linearly with 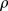 across all prey densities. The encounter rate of signal-modulated predators, on the other hand, is strongly nonlinear in 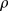 (compare Fig. 3A yellow points to blue triangles). In particular, at low but realistic prey densities (Fig. 3A blue curve), the encounter rate of signal-modulated predators changes sublinearly with changing prey density. This anomalous scaling makes the encounter rate of signal-modulated predators more robust with respect to changes in prey density (see *Implications for coupled population dynamics* below).

### Encounter rates of purely random predators are near-linear in prey density

Predators that used a purely random search strategy encountered prey at a rate that was nearly proportional to prey density (Fig. 3A, yellow circles; 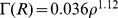; 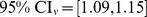). This near-linear scaling held when prey were clustered and also when predators encountered and destroyed multiple prey per search (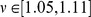). The encounter rate function did not show evidence of multiple scaling regimes (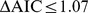 in both clustered and uniform environments with 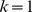 and 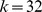). This result mirrors that of particle collision models and other random-walk-based models of organismal search, which all predict that encounter rate is proportional to target density when target density is low.

### Encounter rates of signal-modulated predators change nonlinearly with prey density

Across all densities studied, predators that use sensory data to make movement decisions encounter prey at a higher rate than predators that do not use sensory cues (Fig. 3A, B). Indeed, at low and intermediate densities, signal-modulated predators encounter prey at a rate that can be 5–14 times higher than the encounter rate of random predators (Fig. 3B).

**Figure 4:**
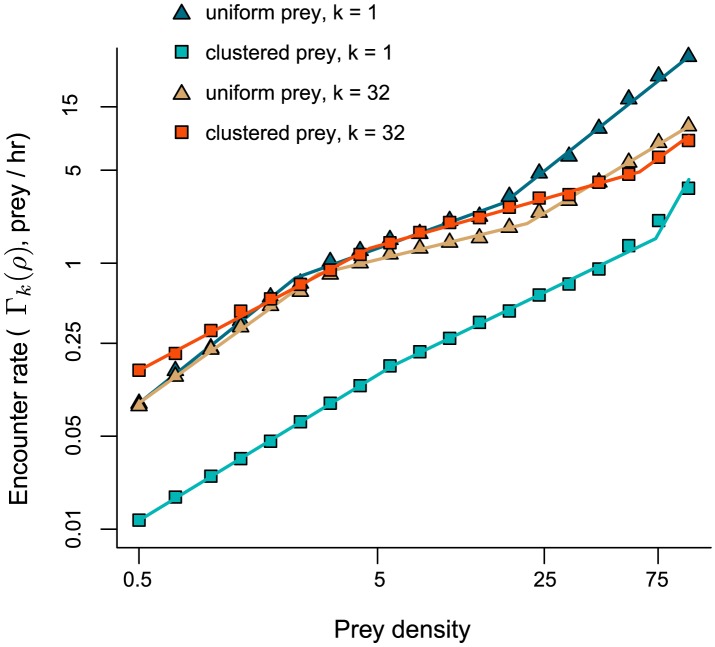
Mean encounter rate of signal-modulated predators in uniform (Poisson) and clustered (preferential attachment) prey environments. Predators encounter and destroy 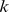 prey items per search. Each point represents mean of 1000 replicate simulations. Parameters as in Fig. 3. Encounter rate is lower in clustered environment with *k* = 1 because clusters are far from one another and it can take predators a long time to locate a cluster. When *k* = 32, encounter rate is higher because the predator can encounter nearby targets after it locates the cluster.

As prey density increases, the encounter rate of signal-modulated predators increases nonlinearly and clearly displays multiple scaling regimes (Fig. 3A, blue triangles; AIC single regime minus AIC three regimes = 682). At the lowest densities, encounter rates increased linearly or superlinearly with prey density. For the particular parameter values explored here, there is a transition to a second scaling regime at 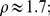 however, the exact transition depends on the length scale of scent detection (Fig. S1). In the second, intermediate regime, which covers low but realistic prey densities, signal-modulated predators encounter prey at a rate proportional to 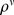, where 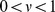. The value of the scaling exponent 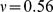, is close the square-root scaling exhibited by the searcher with perfect sensing response. For higher densities, data indicated a third regime, in which encounter rate increased superlinearly with prey density (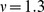) corresponding to conditions in which prey are plentiful and predators do not need to search. The qualitative form of the encounter rate function of signal-modulated predators in a uniform prey environment was preserved when prey were highly clustered, and when predators encountered and destroyed multiple prey items in a single search. Fig. 4 shows that the mean encounter rate after 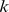 encounters 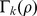 exhibited near-linear regimes at relatively high and low densities, and sublinear regimes at intermediate densities (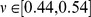 in sublinear regime).

### Sensory response allows predators to encounter nearby targets more frequently

**Figure 5:**
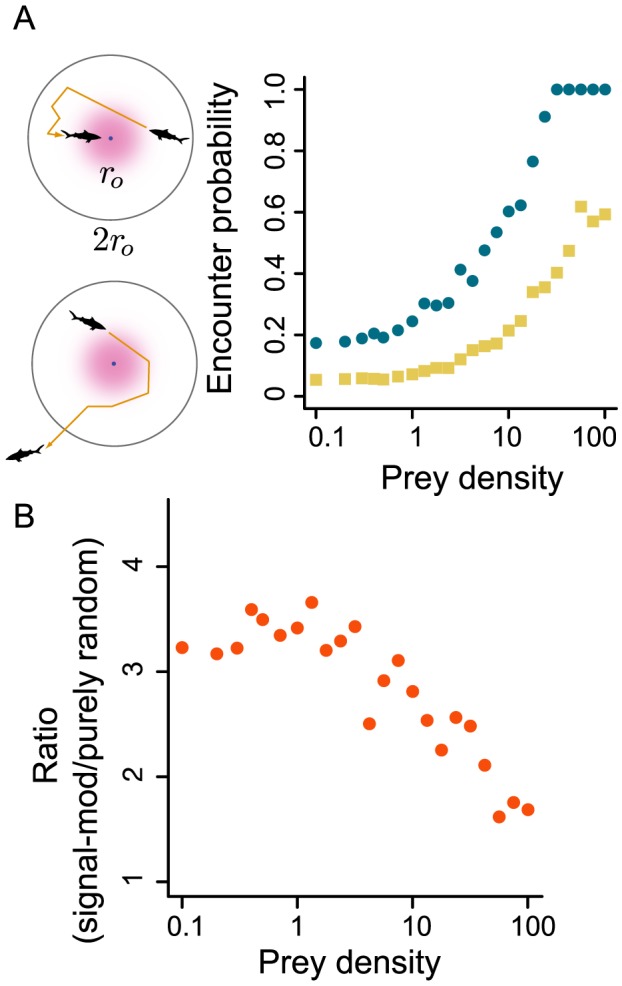
Encounter probabilities of signal-modulated and purely-random predators. A) Encounter probability as a function of target density. Points represent the probability that a purely random predator (yellow squares) or a signal-modulated predator (blue circles) will wander away from a nearby target without encountering it. Parameters as in Fig. 3. Upper diagram shows predator that encounters prey before exiting region of radius 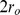. Lower diagram shows predator that exits before encountering prey. B) Ratio of encounter probability of signal-modulated predator to encounter probability of purely random predator.

In addition to engaging in area-retricted search, signal-modulated predators successfully locate nearby prey more frequently than purely random predators (Fig. 5A, upper diagram), which often wander away from nearby prey without encountering them (Fig. 5A, lower diagram). To examine this more rigorously, we isolated all occasions in which a predator came within a distance 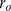 of a prey and defined these as *proximity events*. A proximity event ends when the predator encounters a prey item, or moves to a location that is at least 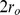 from any prey. We computed the fraction of proximity events that resulted in encounters and defined this as the empirical encounter probability. We chose 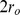 because at that distance, predators have a probability of only 0.05 of measuring a non-zero scent signal in 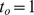 s meaning that a predator beyond this distance is very unlikely to receive any further scent information from prey. Fig. 5A shows that the empirical encounter probability of signal modulated predators (Fig. 5A blue points) is higher for all prey densities, and approaches 1 for prey densities above 10, indicating that signal-modulated predators do not miss nearby targets when density is high. Purely random predators miss nearby prey even as prey density approaches 100 (as 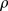 approaches 100, the typical distance between adjacent prey approaches the encounter radius 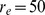 body lengths). At low density, encounter probabilities of both types of predator approach constant values. For the signal-modulated predator, this value is 0.17, similar to the value of 0.23 predicted for a Brownian searcher with constant diffusivity (see Text S1). Fig. 5B shows that this minimum encounter probability is roughly three times higher for signal modulated predators than for purely random predators.

### Implications for coupled population dynamics

Our results demonstrate that the use of sensory information alters encounter rate kinetics, both at the extreme of perfect information and decision-making, and at the other extreme of minimal sensing and rudimentary decision-making. In studies of coupled population dynamics, the encounter rate function is a central component of the functional response, the relationship that couples prey and predator populations. Given the anomalous scaling of encounter rate shown by predators that use sensory information to make movement decisions, a natural question is whether such predator search behavior might affect coupled population dynamics. Here, we explore this question.

Predator-prey dynamics can be modeled by the following system of equations:
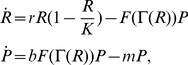
(4)which is a generalized version of the Rosenzweig-MacArthur model [31]. The variables 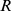 and 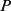 represent the prey and predator densities (number per 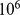 squared predator body lengths) respectively. The intrinsic growth rate of the prey population is given by the parameter 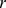, and prey growth is limited by the carrying capacity, 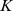, in the absence of predators. The predator population is assumed to die at rate 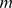 and the parameter 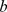 is a measure of predator energy conversion efficiency. All time rates are on a per day scale.

To relate our encounter rate findings to coupled population dynamics, we must translate the encounter rate 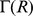 into a long-term functional response 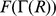. This is necessary because search and reproduction take place on distinct time scales (e.g., hours versus years, respectively). We consider a scenario that reflects the general theme of the work presented here: a regime where prey are sparse, but rare encounters are sufficient to sustain a predator. We assume that predators undertake a succession of hunting expeditions each day. The predator engages in 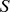 hunts per day, each lasting a period 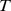 before the predator relents. The hunt is assumed to end if a prey is captured. Therefore the number of consumption events in a given day, which we denote 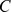 can be written as the sum 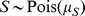 of indicator functions 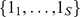 which take the value one or zero depending on whether the corresponding search expedition is successful. The success probabilities 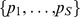 depend on the encounter rate 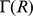 and the respective search durations 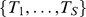. Adopting the simplest assumption for the search distribution, we take each 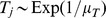. Then, treating the encounter process as a Poisson process with rate parameter 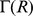, it follows that, given the value of 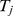, the success probability of the *j*th hunt is 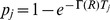. Using Wald's equation, a simple calculation reveals the following form for the functional response:
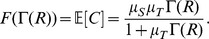
Note that this form closely resembles the Holling type II functional response [32]. Note also that it is increasing in prey density, concave down, and satisfies
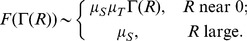



We now proceed to study the effect of the form of 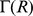 on the outcome of predator-prey dynamics. The mathematical structure of (4) is more readily apparent if we introduce the functions 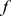 and 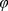 to generalize the autonomous growth function and functional response, respectively. The function 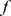 should be zero when the prey density 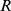 is equal to zero or 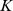, differentiable, and concave down everywhere. We take 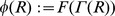, and writing 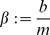 we have
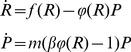
(5)We see that if 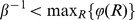 then 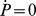 when 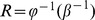. Moreover, if 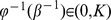 there exists a *coexistence* fixed point 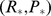 with 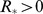 and 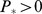, where
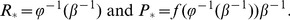
(6)Under suitable conditions, which biologically plausible forms of 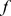 and 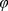 will generally satisfy, this fixed point is unique. In Text S1 we analyze the stability of the coexistence fixed point for systems with encounter rates of the form 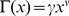 where 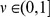. We show that there is a critical value 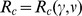 such that if 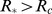 the coexistence fixed point is stable. Otherwise, it is unstable; however, numerical studies indicate the presence of a stable limit cycle that contains the fixed point. Notably, for values of 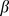 that lead to very low values of 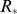, the coexistence fixed point is unstable. This is true for all models considered here, including those with a linear encounter rate function. This fundamental instability is due to the nonlinear nature of the Holling Type II form we use to translate the encounter rate into functional response.

**Figure 6:**
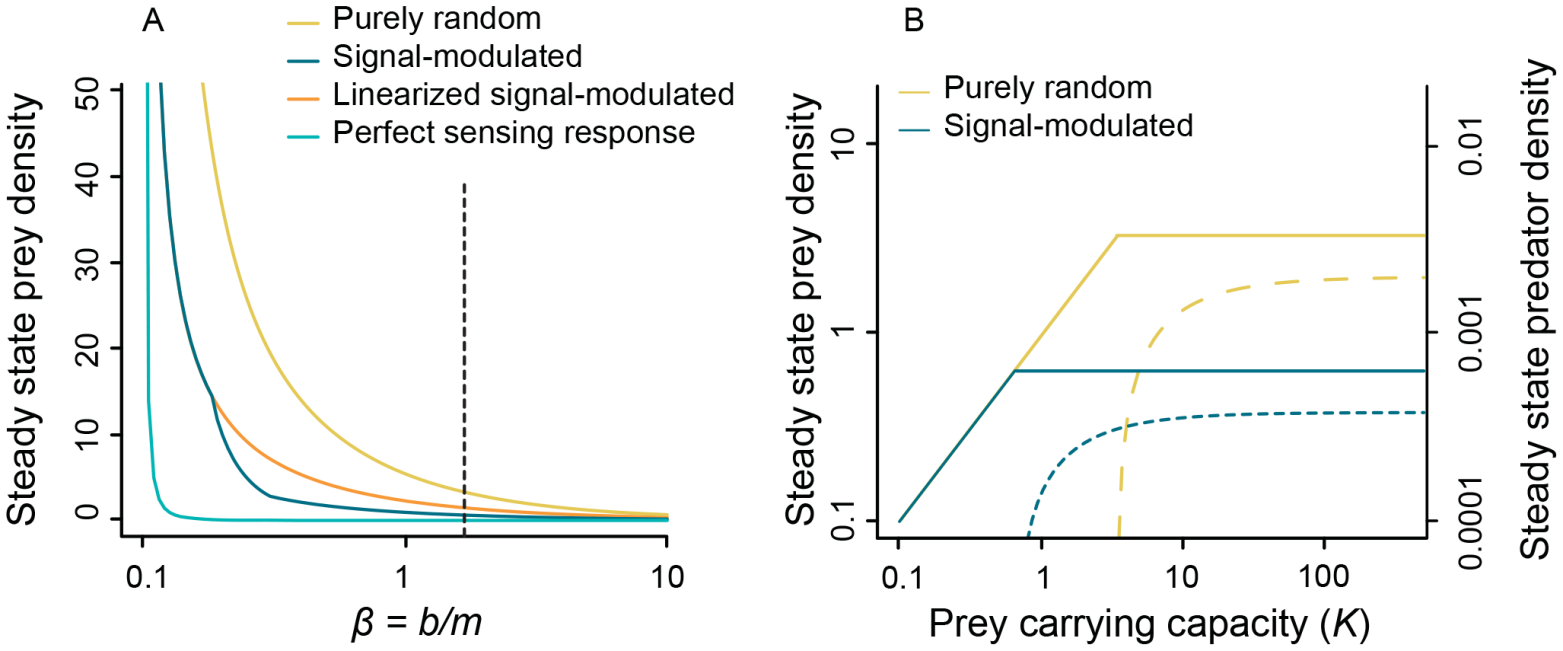
Equilibrium population densities of predators with different search strategies. A) Steady state prey density as a function of the ratio of predator conversion efficiency to predator mortality rate (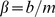). Predators with perfect sensing and response and signal-modulated predators reduce prey density to lower values than purely random predators. The “linearized signal-modulated” is identical to the signal-modulated predator in the high density regime but has linear encounter rate in the low density regime (see text). B) Equilibrium densities of prey (solid lines, left ordinate) and purely random (dashed line, right ordinate) and signal-modulated predators (dotted line, right ordinate) for fixed value of 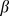 (Indicated by black vertical dashed line in panel A). Parameter ranges chosen based on reasonable rates and constants from lion-wildebeest interactions [25],[36]: 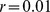 (prey offspring per prey per day), 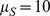 (searches per day), 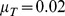 (days), 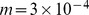 (predators per day), 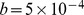 (predator offspring per predator per day). We used encounter rate functions described by the fitted curves in Fig. 3A for purely random and signal-modulated predators.

To explore how the form of the encounter rate function affects the outcome of coupled population dynamics, we parameterize the population model described above for the sparse prey regime (Fig. 6). This analysis demonstrates several differences between population dynamics involving sensory predators and more traditional models that assume a linear encounter rate function. First, predators that use sensory data deplete prey to lower densities than predators that search randomly. The ability to deplete prey to low levels is a critical trait in ecological dynamics; for example, R^*^ theory posits that a species' competitive ability is determined by its ability to deplete resources and persist when resources are rare [4]. Fig. 6A shows the steady state density of prey as a function of the ratio of predator conversion efficiency 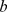 to predator mortality rate 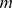. Both signal-modulated predators and predators with perfect sensing and decision-making reduce prey density to lower levels than do purely random predators (Fig. 6A, blue and cyan curves are below yellow curve). To demonstrate the extent to which the nonlinearity in the encounter rate function of signal-modulated predators contributes to this pattern, we added a “linearized signal-modulated encounter rate” (Fig. 6A orange curve), which matches the signal-modulated predator for prey densities above 20, but remains linear for lower densities. Signal-modulated predators (Fig. 6A, blue curve) reduce prey density well below that of the linearized analogue (Fig. 6A, orange curve) illustrating the substantial effect of the nonlinearity in the encounter rate function. We note that throughout the depicted regime, the coexistence fixed point is unstable, but with a containing stable limit cycle.

A second important observation is that, for a given value of the parameter 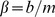, signal-modulated predators persist at lower prey carrying capacity 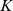 than purely random predators, and predator density of signal-modulated predators is less sensitive to variation in 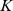. Fig. 6B shows steady state density of predators (dashed lines) and prey (solid lines) as a function of prey carrying capacity. Steady-state prey density does not depend on carrying capacity until 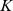 becomes so low that predators no longer persist. Below this point, prey steady state density is equal to 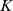. Steady state density of purely random predators rapidly decays to zero below 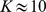. Steady state density of signal-modulated predators, on the other hand, remains insensitive to carrying capacity for 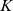 greater than approximately 2.

## Discussion

Our results demonstrate that the use of information about the position of targets fundamentally alters the relationship between encounter rates and target density. Not only do predators that use sensory information encounter prey more often, but they are less sensitive to changes in prey density. This is true even when sensory cues contain a minimal amount of information about target locations, and searchers do not remember past signals. This increased robustness provides an ecological mechanism through which sensory response may allow predators to cope with fluctuations in prey density. Moreover, it can alter coupled population dynamics. These findings are robust to a range of assumptions about target distribution, capture behavior, and the length over which searchers detect scent signals (Text S1).

Reaching a general understanding of the effect of sensory data on species encounter rates is challenging. Searching organisms collect a wide variety of sensory data and biologists do not know, in general, how they use these data to make decisions [17]. Here, we have taken the approach of studying two extreme cases of the collection and use of sensory data. In the extreme of perfect sensing and response, predators encounter prey at a rate proportional to prey density to the 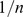 power at low prey density (where 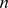 is the dimension of the search environment), rather than exhibiting the linear scaling predicted by models of purely random search. At the opposite extreme, when we perturb purely random search behavior by introducing a very limited capacity for sensing and decision-making based on a noisy, directionless signal, the encounter rate function immediately departs from the linearity expected when predators move without using information [2],[5],[6]. This observation has immediate implications for classical population models, where the encounter rate informs the functional response. The functional responses that are most commonly used in predator-prey models are either linear in prey density (e.g., Holling type I), or nonlinear and concave down at high densities to incorporate effects of satiation and handling time when prey density is high (e.g., Holling type II). These forms are linear when prey density is low. By contrast, we argue that in the important regime where prey are rare and predators must search for them, the functional response is *nonlinear* and *concave down*. This change in the form of the functional response makes predators less sensitive to changes in prey density and can alter the outcome of predator-prey interactions by allowing predators to deplete prey to lower levels and persist with prey over a broader range of prey carrying capacity.

Our work suggests several ways to better integrate experiments with models of encounter rates. For example, we suggest that encounter rate and functional response of predators should be nonlinear at low prey densities. Yet, most experimental studies of encounter rates and functional response measure rates at high prey density, where handling time and predator satiation determine the shape of the rate function (but see [33],[34], which show concave down encounter rate functions in hunting fish and birds as we predict). Data from carefully designed experiments are needed to determine the most appropriate forms of encounter rate functions and functional responses at low prey density. Distinguishing “high” from “low” prey density is not arbitrary; rather, high and low density regimes are determined by the length scale of predator-prey encounters (e.g., predator striking distance) and by the length scale of the propagation of sensory signals. Future experimental work should evaluate the scaling of encounter rate with 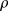 when the typical distances between prey are similar to or greater than the distance at which predators can acquire sensory cues from prey.

Finally, we note that nonlinearity of the encounter rate function depends on the ratio of the length scale of sensory signal transmission to the length scale at which encounters occur. When predators can only detect prey that are very nearby (i.e. detection distance/encounter distance 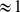), sensory information does not strongly affect search performance [12], and mass action kinetics may provide a reasonable description of encounter rate kinetics. For example, in predator-prey interactions at low Reynold's number, cruising predators may still use sensory information to make movement decisions, yet the length scales associated with prey detections can be very short (e.g., less than one predator body length) relative to the distances between adjacent prey [35].

Our results show that introducing a response to even relatively information-poor, noisy sensory signals qualitatively alters the relationship between predator-prey encounter rate and prey density in many biologically plausible scenarios. Behaviors such as area-restricted search emerge naturally from our model of search behavior, even in the absence of signal gradients, complex signal processing, and memory of past signal and target encounters [12]. The framework we introduce here can be used to understand the connection between information and the encounter rates that are so critical to many core concepts in biology.

## Supporting Information

Figure S1
**Sublinear regime and olfaction radius.** Breakpoint between low density linear regime and sublinear regime as a function of the predator olfaction radius 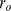.(EPS)

Figure S2
**Non-linear scaling of encounter rate when intrinsic movement distribution is diffusive.** Mean encounter rate of signal-modulated predators in uniform (Poisson) and clustered (preferential attachment) prey environments when intrinsic movement distribution leads to diffusive behavior (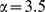). Predators encounter and destroy 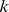 prey items per search. Each point represents mean of 1000 replicate simulations. With the exception of 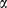, all parameters as in Fig. 3 (main text).(EPS)

Text S1 **Supporting derivations, calculations, and parameter values for search and population models.** Detailed description of scent propagation model, discussion of encounter probability and encounter rates for perfect sensing and response model, and robustness of results to variation in assumptions about predator behavior.
(PDF)
