# Supplementary material for: Sensory Information and Encounter Rates of Interacting Species
Source: PLoS Comput Biol. 2013 Aug 15;9(8):e1003178. doi: 10.1371/journal.pcbi.1003178 (PMC3744405; doi:10.1371/journal.pcbi.1003178)
Supplement: Text S1 — Supporting derivations, calculations, and parameter values for search and population models. Detailed description of scent propagation model, discussion of encounter probability and encounter rates for perfect sensing and response model, and robustness of results to variation in assumptions about predator behavior. (PDF) [file pcbi.1003178.s003.pdf]

Text S1: Sensory information and encounter rates of interacting species.

Andrew M. Hein and Scott A. McKinley

1 Scent propagation

We model scent propagation in turbulence as packets that appear at the prey position $\mathbf{x_0}$ according to a Poisson arrival process and move as a Brownian motion. From the predator's perspective, this is equivalent to encountering a random number of units of scent, $H \sim \text{Pois}(t_o R(|\mathbf{x}-\mathbf{x_o}|))$, at its location $\mathbf{x}$ during a scanning phase of length t_o, where R is the rate of scent arrival. Denoting $\ell = |\mathbf{x}-\mathbf{x_0}|$, under these assumptions, the likelihood of h encounters is $P(H = h|\ell) = [t_o R(\ell)]^h e^{-t_o R(\ell)}/h!$. To derive $R(\ell)$, let $u(\mathbf{x})$ represent the mean concentration of scent at predator position $\mathbf{x}$ emitted by a prey item located at position $\mathbf{x_0}$. The steady-state diffusion process without advection is described by

$$0 = D\Delta u(\mathbf{x}) - \mu u(\mathbf{x}) + \lambda\delta(\mathbf{x}_0) \tag{S1}$$

where D represents the combined molecular and turbulent diffusivity ($m^2 s^{-1}$), μ represents the rate of dissolution of scent patches (s^{-1}), and λ represents the rate of scent emission at the prey (s^{-1}). In two dimensions, the rate of scent patch encounters by a predator of linear size a located at $\mathbf{x}$ is given by $R(\ell) = \frac{2\pi D}{-\ln(a\psi)} u(\ell)$ where $\psi = \sqrt{\frac{\mu}{D}}$. This implies

$$R(\ell) = 2\frac{\lambda K_0(\psi\ell)}{-\pi\psi\ln(\psi a)} \tag{S2}$$

where K_0 represents a modified Bessel function of the second kind. Two terms are sufficient to characterize the scent environment: the typical propagation length r_o, which corresponds to the distance at which a predator will register on average one unit of scent per scanning period, and the expected number of encounters per unit t_o at a distance of one body length from the prey.

2 Dependence of regime break on scent signal propagation length, and dependence of results on properties of the intrinsic movement distribution

To determine whether the prey density at which linear regimes in the encounter rate function transitioned to nonlinear regimes depended on the length scale of predator scent detection, we repeated simulations to compute $\Gamma(\rho)$ over a range of values of the olfaction radius r_o. Figure S1 shows that the prey density at which the linear regime transitions to a sublinear regime decreases as r_o increases. Thus, when prey scent propagates over a longer

distance, the sublinear scaling of encounter rate persists to lower prey density.

As described in the main text, many studies have disputed whether organisms use search strategies that can be described as random walks in which the lengths of movements are drawn from statistical distributions with heavy tails, resulting in so called "Lévy walk" behavior. In the main text, we adopt a distribution for the intrinsic movements $\gamma(\theta, \ell)$, that has a power law tail with the exponent $\alpha = 2$, which will lead to superdiffusive Lévy walk behavior. However, as we show in the main text and as has been shown in past work [1], such behavior does not lead to nonlinear scaling of the encounter rate function with prey density in the absence of sensory signals. When signals are incorporated, the effect of signal data can dominate the choice of intrinsic movement strategy such that, regardless of whether a predator uses a heavy tailed intrinsic movement distribution, or one that decays more quickly, the realized movement behavior is very similar [2]. Still, to ensure that our results were not determined by the use of a superdiffusive intrinsic movement strategy, we repeated all search simulations after changing the value of the Pareto exponent α to 3.5. For values of α above 3, the variance of the Pareto distribution is finite and the long-term behavior is diffusive rather than superdiffusive. Figure S2 shows that multiple scaling regimes including a sublinear regime at low-intermediate densities emerge when predators use sensory data ($\nu \in [0.41, 0.69]$ in sublinear regime). The qualitative conclusion that signal-modulation leads to the emergence of a sublinear regime in the encounter rate function matches results shown in the main text (Figures 3 and 4). Our results do not depend on whether the intrinsic movement distribution is diffusive or superdiffusive.

3 Encounter rate of a predator with perfect sensing and response, and non-zero encounter radius

Suppose that a predator is located at the origin of an n-dimensional environment containing prey distributed according to a Poisson spatial process with intensity ρ. We calculate the expected distance to the nearest prey to reveal the general relationship that the expected encounter rate scales with prey density ρ as $\rho^{1/n}$. Let ℓ_{np} denote this distance to the nearest prey. Because we have assumed the prey are distributed according to a spatial Poisson process, the probability that there are no prey within a radius r of the predator is given by $\mathbb{P}\left\{\ell_{\text{np}} > r\right\} = e^{-\rho|B_r^{(n)}|}$ where $|B_r^{(n)}|$ is the volume of an n-dimensional ball of radius r. Defining $C_n = \frac{\pi^{n/2}}{\Gamma\left(1+\frac{n}{2}\right)}$, where $\Gamma(s) := \int_0^\infty y^{s-1}e^{-y}dy$ is the gamma function, we note that $|B_r^{(n)}| = C_n r^n$.

Now, let $\ell_e := \max(0, \ell_{\text{np}} - r_e)$ denote the distance the omniscient predator has to travel to reach the encounter

radius of the nearest prey. Using the formula for expectation and then integrating by parts, we can write

$$\begin{aligned}\mathbb{E}\left[\ell_e\right] &= -\int_{r_e}^{\infty}(r-r_e)\frac{d}{dr}\mathbb{P}\left\{\ell_{\mathrm{np}}>r\right\}dr \\ &= -(r-r_e)\mathbb{P}\left\{\ell_{\mathrm{np}}>r\right\}\Big|_{r=r_e}^{\infty}+\int_{r_e}^{\infty}\mathbb{P}\left\{\ell_{\mathrm{np}}>r\right\}dr \\ &= \int_{r_e}^{\infty}\exp\left(-\rho C_n r^n\right)dr\end{aligned}$$

Under the substitution $y=\rho C_n r^n$, this integral becomes

$$\mathbb{E}\left[\ell_e\right]=\frac{1}{\rho^{\frac{1}{n}}}\frac{1}{nC_n^{\frac{1}{n}}}\int_{\rho C_n r_e^n}^{\infty}y^{\frac{1}{n}-1}e^{-y}dy=\frac{1}{\rho^{\frac{1}{n}}}\frac{1}{nC_n^{\frac{1}{n}}}\left(\Gamma\Big(\frac{1}{n}\Big)-\gamma\Big(\frac{1}{n},\rho C_n r_e^n\Big)\right)$$

where $\gamma(s,x):=\int_0^x y^{s-1}e^{-y}dy$ is the incomplete gamma function. The leading order terms of the power series expansion for the incomplete gamma function are $\gamma(s,x)=e^{-x}\Big(x^s\frac{\Gamma(s)}{\Gamma(s+1)}+x^{s+1}\frac{\Gamma(s)}{\Gamma(s+2)}+O(x^{s+2})\Big)$, which leads to the expansion

$$\mathbb{E}\left[\ell_e\right]=\rho^{-\frac{1}{n}}\left(\frac{\Gamma(\frac{1}{n})}{nC_n^{\frac{1}{n}}}\right)+\exp\big(-\rho C_n r_e^n\big)\left[\frac{\Gamma(\frac{1}{n})}{\Gamma\big(1+\frac{1}{n}\big)}+O(\rho)\right] \tag{S3}$$

The expected time between encounters is then $\mathbb{E}\left[\ell_e\right]/v$ where v is the velocity of the predator. The encounter rate is then computed asymptotically by looking at the number of of encounters as of time t, $N(t)$, that occur per unit time. The Renewal Theorem reveals that

$$\Gamma(\rho)=\lim_{t\to\infty}\frac{N(t)}{t}=\frac{v}{\mathbb{E}\left[\ell_e\right]}=\frac{v\rho^{\frac{1}{n}}}{K+o(\rho^{\frac{1}{n}})}. \tag{S4}$$

where K is a constant that depends only on N. In the specific case of two dimensions, the random distance between the predator and the nearest prey is given by the Rayleigh distribution, which has density $p(\ell)=2\rho\pi\ell e^{-\rho\pi\ell^2}$. We then observe that

$$\mathbb{E}\left[\max(\ell_{\mathrm{np}}-r_e,0)\right]=\int_{r_e}^{\infty}(\ell-r_e)p(r)dr=\frac{1}{2\sqrt{\rho}}\left(1-\operatorname{erf}(r_e\sqrt{\pi\rho})\right),$$

where $\operatorname{erf}(\mathrm{x})=\frac{2}{\sqrt{\pi}}\int_0^x e^{-z^2}dz$.

To observe the square root scaling, simply note that $\operatorname{erf}(x)\to 0$ as $x\to 0$. It follows that $\Gamma(\rho)\sim 2\sqrt{\rho}/v$ in this regime. For larger ρ, the error function behaves like $1-\operatorname{erf}(x)=\frac{e^{-x^2}}{x\sqrt{\pi}}+O\big(x^{-3}e^{-x^2}\big)$ so that, to leading order,

$$\Gamma(\rho)=\frac{2\sqrt{\rho}}{v(1-\operatorname{erf}(r_e\sqrt{\pi\rho}))}\sim\frac{2\pi r_e}{v}\,\rho\, e^{r_e^2\pi\rho}.$$

Because r_e and ρ are small in the parameter regime of interest, there is a range of ρ, roughly from 10 to 100, for which encounter rate scales roughly linearly with ρ (i.e. $e^{r_e^2\pi\rho}\approx 1$). This is seen in Figure 1 in the main text. As

ρ becomes large, the scaling is exponential; however, for the cases of interest here (i.e. relatively low prey density), the exponential regime is not relevant.

4 Encounter probabilities in the sparse regime

When prey density is very sparse, each prey exists essentially in isolation. This is why the empirically observed probability of encounter with nearby prey stabilizes for low prey density (see Fig. 5, main text) In this section, we aim to estimate this probability in the sparse prey regime. As described in the main text, a proximity event begins when the predator comes within a radius r_o of the prey. We pick this length because the expected signal size is one unit and the probability of the signal being nonzero is nontrivial (0.63). If the predator happens to take steps away from the prey it may reach a distance where it is exceedingly unlikely that another signal will be received from that prey. At such a distance, we consider the interaction to have ended without an encounter (and hence subsequent capture). To find an analytical estimate for this sparse regime scaling, we propose the following problem from classical probability theory. We approximate predator motion by a Brownian motion that has diffusivity D. The prey is located at the origin and the predator is located uniformly at random among all points that are a distance distance r_o from the origin. We compute the probability that the predator hits a circle of radius r_e before exiting a concentric circle of radius zr_o. This is an exactly solvable problem. The predator's radial distance from the origin evolves according to a Bessel process $R(t)$ that satisfies following Itô form stochastic differential equation [3]

$$dR(t) = \frac{D}{R(t)}dt + \sqrt{2D}dW(t), \qquad R(0) = r_o.$$

The probability that this process hits the level r_e before zr_o is given by the solution to the ODE

$$Dp''(r) + \frac{D}{r}p'(r) = 0$$

with $p(r_e) = 1$ and $p(zr_o) = 0$. The general solution is readily shown to be

$$p(r) = \frac{\ln(zr_o) - \ln(zr)}{\ln(zr_o) - \ln(r_e)}$$

which, plugging in the initial condition $R(0) = r_o$ yields

$$p(r_o) = \frac{\ln z}{\ln z + \ln(\frac{r_o}{r_e})}. \tag{S5}$$

The approximation is successful because in the presence of signal, the likelihood function in the Bayesian update, Equation (1), truncates the power law tail of the default Pareto distribution. Random walks with exponential jump

tails are diffusive in character, meaning that Brownian motion can give a somewhat authentic scaling in r_o and r_e. Furthermore, note that the hitting probability for Brownian motion is insensitive to its diffusivity, meaning we do not have to attempt to tune the Brownian motion to match the imperfectly sensing predator. On the other hand, the effective diffusivity of the imperfectly sensing predator is certainly state dependent because larger signal magnitudes lead to shorter jump lengths. A further defect of the Brownian approximation is that it will always *overestimate* the encounter probability because the imperfect predator will occasionally experience zero signal when somewhat distant from the prey. This means imperfectly sensing predators will occasionally sample from the jump distribution with heavy tail and increase its chance of escape before reaching the prey.

5 Stability Analysis for the Predator-Prey model

In order to determine the local stability of the coexistence fixed point, we compute the Jacobian and evaluate at (R_*, P_*),

$$J(R_*, P_*) = \begin{pmatrix} f'(R_*) - f(R_*)\frac{\varphi'(R_*)}{\varphi(R_*)} & -\varphi(R_*) \\ m\beta f(R_*)\frac{\varphi'(R_*)}{\varphi(R_*)} & 0 \end{pmatrix}. \tag{1}$$

In the above formulation we have used the fact that, when written in terms of R_*, the predator fixed point value is $P_* = \frac{f(R_*)}{\varphi(R_*)}$. The stability of this system depends on whether the trace occur when the trace $T(R_*) = f'(R_*) - f(R_*)\frac{\varphi'(R_*)}{\varphi(R_*)}$ is positive (unstable) or negative (stable). As we will see, for relevant choices of f and φ, there is a critical prey density R_c that satisfies

$$\frac{f'(R_c)}{f(R_c)} = \frac{\varphi'(R_c)}{\varphi(R_c)}. \tag{2}$$

To understand the bifurcation more clearly, we consider the special choices $f(x) = rx(1-x/K)$ and $\varphi(x) = \frac{\mu_S\mu_T\Gamma(x)}{1+\mu_T\Gamma(x)}$. We further suppose that $\Gamma(R) = \gamma R^\nu$ for some $\gamma > 0$ and $\nu \in (0, 1]$ in a neighborhood of the prey density fixed point R_*. Checking whether the trace is positive reduces to checking whether

$$\frac{K - 2R_*}{K - R_*} > \frac{R_*\Gamma'(R_*)}{\Gamma(R_*)(1 + \mu_T\Gamma(R_*))} = \frac{\nu}{1 + \mu_T\gamma R_*^\nu}. \tag{3}$$

A quick calculation shows that there is a unique R_c and for all $R_* > R_c$, the coexistence steady state is stable. For all $R_* < R_c$, the fixed point is unstable; however numerical studies demonstrate there is stable limit cycle that contains the unstable coexistence equilibrium. For the form of functional response considered here (which is a Holling type II functional response when encounter rate is linear in prey density), *all encounter rate models* yield dynamics with a region of instability. Indeed, when the encounter rate is linear, $\Gamma(x) = \gamma x$ for some γ, which has

the units of number of prey encountered per hour, $R_c = \frac{1}{2}K - \frac{1}{2\gamma\mu_T}$ revealing that the region of instability exists even for predators with linear encounter rate functions.

References

[1] James A, Pitchford JW, Plank MJ (2010) Efficient or inaccurate? analytical and numerical modelling of random search strategies. Bull Math Biol 72: 896-913.

[2] Hein AM, McKinley SA (2012) Sensing and decision-making in random search. Proc Natl Acad Sci USA 109: 12070–12074.

[3] Øksendal B (2010) Stochastic Differential Equations: An Introduction with Applications. Springer, Berlin.
